# Association between polymorphisms of arachidonate 12-lipoxygenase (*ALOX12*) and schizophrenia in a Korean population

**DOI:** 10.1186/1744-9081-6-44

**Published:** 2010-07-14

**Authors:** Tae Kim, Hak-Jae Kim, Jin Kyung Park, Jong Woo Kim, Joo-Ho Chung

**Affiliations:** 1 Department of Neuropsychiatry, School of Medicine, Kyung Hee University, Seoul, Korea; 2 Department of Psychiatry, Harvard Medical School-VA Boston healthcare system, West Roxbury, USA; 3 College of Medicine, Soonchunhyang University, Chunan, Korea; 4 Department of Psychiatry, Kyung Hee University East-West Neo Medical Center, Seoul, Korea; 5 Kowhang Medical Research Institute, School of Medicine, Kyung Hee University, Seoul, Korea

## Abstract

Arachidonic acid (AA), an essential polyunsaturated fatty acid, is one of the major components of neural membranes, which show an altered phospholipid composition in schizophrenia. Arachidonate 12-lipoxygenase (*ALO*X12), an important enzyme, metabolizes AA to 12-HPETE, which affects catecholamine synthesis. However, research has yet to show the genetic association between ALOX12 and schizophrenia. Therefore, we investigated single nucleotide polymorphisms (SNP) of the *ALOX12 *gene in schizophrenia, recruiting patients with schizophrenia (n = 289) and normal controls (n = 306) from a Korean population. We selected three SNPs (rs1126667, rs434473, and rs1042357) of the *ALOX12 *gene and genotyped them by direct sequencing. We reviewed the schizophrenic patients' medical records and assessed them clinically using the Brief Psychiatric Rating Scale (BPRS), the Scale for the Assessment of Negative Symptoms (SANS), and the Operational Criteria Checklist (OPCRIT). Then we statistically analyzed the genetic associations between the SNPs and schizophrenia, finding a genetic association between both rs1126667 and rs1042357 and schizophrenia, in the recessive model (p = 0.015 and 0.015, respectively). We also found an association between rs434473 and negative symptoms, defined through a factor analysis of the OPCRIT data (p = 0.040). Consequently, we suggest that SNPs of the *ALOX12 *gene might be associated with schizophrenia and negative symptoms in this Korean population. These weak positives require additional study.

## Findings

Essential polyunsaturated fatty acids (EPUFAs) are major components of neural membranes. These membranes show an altered composition in schizophrenia patients [1]. Arachidonic acid (AA) and docosahexaenoic acid (DHA) constitute 80-90% of EPUFAs in neuronal tissue, but there is reduced AA in schizophrenia [2]. The enzyme *ALO*X12 oxygenates the C-12 of arachidonic acid, producing 12-hydroperoxy-5, 8, 10, 14- eicosatetraenoic acid (12-HPETE) [3], which, research has shown, inhibits protein kinase II [4]. Protein kinase II phosphorylates tyrosine hydroxylase, which is the rate-limiting enzyme in catecholamine synthesis [5]. Accordingly, 12-HPETE might affect dopamine synthesis. In addition, 12- HPETE inhibits neurotransmitter release per se [6]. However, no research has yet shown the association between ALOX12 and schizophrenia. Therefore, we hypothesized genetic variances of the *ALOX12 *gene might be associated with schizophrenia. To test this hypothesis, we investigated the single nucleotide polymorphisms (SNPs) of the ALOX12 gene in schizophrenia patients.

We recruited patients meeting the Diagnostic and Statistical Manual of Mental Disorders, Fourth Edition (DSM-IV) criteria for schizophrenia [7], resulting in a study population consisting of 289 Korean patients with schizophrenia [189 men and 100 women; age 42.85 ± 10.84 (mean ± S.D., in years)]. We also recruited 306 Korean control subjects (148 men and 158 women; age 36.06 ± 6.79). We reviewed the medical records of each patient and assessed the schizophrenia patients using the Brief Psychiatric Rating Scale (BPRS), the Scale for the Assessment of Negative Symptoms (SANS), and the operational criteria (OPCRIT) checklist [8]. All studies were carried out according to the guidelines of the Declaration of Helsinki [9]. Written informed consent was obtained from each subject, and the study was approved by the Institutional Review Board of Kyung Hee University Hospital, Seoul, Republic of Korea. We selected three coding SNPs with validated heterozygosity (>0.2; http://www.ensembl.org, http://www.ncbi.nlm.nih.gov/SNP, http://www.hapmap.org): coding region SNPs rs1126667, rs434473, and rs1042357 (SNP database, BUILD 129, Table 1). We used direct sequencing to conduct SNP genotyping and amplified Genomic DNA was using the following primers for each SNP: rs1126667 (sense, 5'- TCAACTCAGAGAGGCCTTGAGAA-3'; antisense, 5'- AAGTGAGGA AGTGCCATCAGGTG-3'; 622 bp), rs434473 and rs1042357 (sense, 5'- CTCC TTCACATTCCACCACCATC-3'; antisense, 5'- GTGAGTGAAGAGGAGACTGT CTC-3'; 625 bp). Then, we sequenced the samples using an ABI Prism 377 automatic sequencer (PE Applied Biosystems, Foster City, CA, USA) and analyzed sequence data using SeqMan II software (DNASTAR Inc., Madison, WI, USA). The genotyping completion rates of our samples were 96.4% (rs1126667), 95.0% (rs434473), and 98.1% (rs1042357). We excluded samples with 1 or more misread or unreadable SNPs. Therefore, 95.0% of the original samples were analyzed. We analyzed Hardy-Weinberg equilibrium (HWE) and genotype frequencies using SNPStats [10] and used multiple logistic regression models to calculate odds ratios (OR), 95% confidence intervals (CI), and corresponding p values; to control age and gender as covariables; and to analyze the association between SNPs and schizophrenia. To calculate the power of the sample size, we used a genetic power calculator http://pngu.mgh.harvard.edu/~purcell/gpc. In our case-control study, the powers were 0.8142 (rs1126667; effective sample size; number of cases for 80% power = 292), 0.8105 (rs434473, n = 292), and 0.8141 (rs1042357, n = 296). However, the sample power in subgroup-divided schizophrenia was not sufficient (data not shown). We also tested clinical variables, including OPCRIT checklist data. The significance level for all statistical tests was 0.05, and we applied Bonferroni corrections for multiple tests in the association study (number of tests = 18).

**Table 1:** Genotype distribution of SNPs of the *ALOX12 *gene among schizophrenia patients in a Korean population.

SNP	Amino acid	Genotype	Control N = 306		Schizophrenia N = 289		Model	OR	95% CI		p
								
			Freq.	%	Freq.	%			LCL	UCL	
rs1126667	R261Q	G/G	96	31.4	77	26.6	Codominant	1.09	0.75	1.59	0.048
(Exon 6)		A/G	151	49.4	132	45.7	Dominant	1.26	0.88	1.80	0.200
		A/A	59	19.3	80	27.7	Recessive	1.60	1.09	2.35	0.015*

rs434473	S322N	A/A	96	31.4	78	27.0	Codominant	1.09	0.74	1.58	0.073
(Exon 8)		A/G	153	50.0	135	46.7	Dominant	1.24	0.87	1.76	0.240
		G/G	57	18.6	76	26.3	Recessive	1.56	1.06	2.30	0.025*

rs1042357	T364T	C/C	95	31.1	77	26.6	Codominant	1.07	0.73	1.57	0.050*
(Exon 8)	A/C	152	49.7		132	45.7	Dominant	1.24	0.87	1.77	0.240
		A/A	59	19.3	80	27.7	Recessive	1.60	1.09	2.35	0.015*

ALOX12, arachidonate 12-lipoxygenase; R, arginine; Q, glutamine; S, serine; N, asparagine; T, threonine; Freq, frequency; OR, odds ratio; CI, confidence interval; LCL, lower confidence limit; UCL, upper confidence limit; p, probability value.

*These values are not significant after the Bonferroni correction (number of tests = 18).

**Figure 1:**
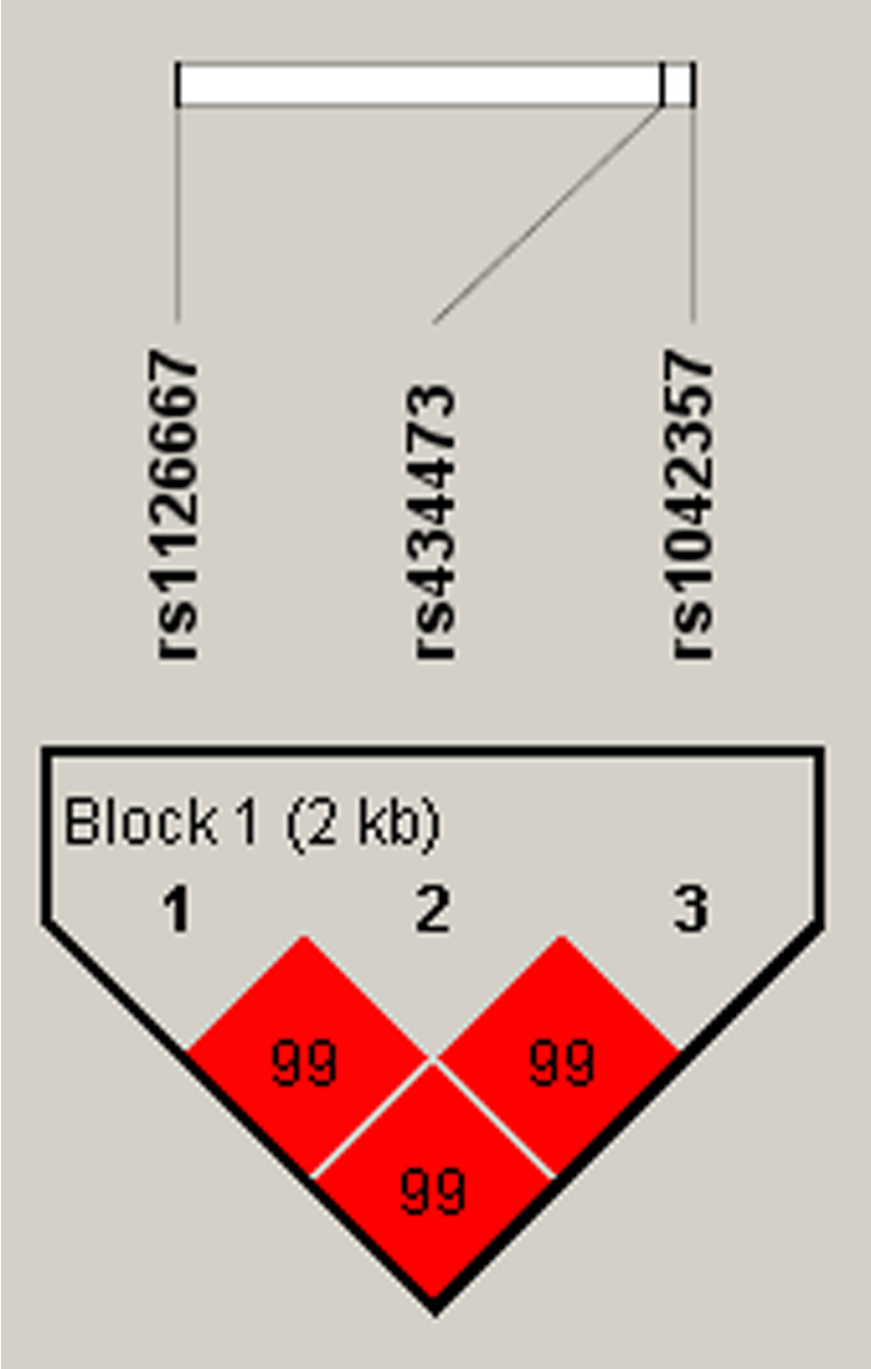
**Linkage disequilibrium (LD) block of *ALOX12 *gene**. LD coefficient (|D'|) and LD blocks among *ALOX12 *SNPs. Block consists of rs1126667, rs434473, and rs1042357.

We found no deviation from the Hardy-Weinberg equilibrium for the selected SNPs. The SNPs rs1126667 and rs1042357 showed significant associations in the recessive model after Bonferroni correction (p = 0.015, OR = 1.60, 95% CI = 1.09-2.35; p = 0.015, OR = 1.60, 95% CI = 1.09-2.35, Table 1). We found no genetic association with gender, schizophrenia subtypes, BPRS, SANS, or any of the OPCRIT items. However, when we categorized the OPCRIT items into four symptom subgroups (excitement, negative symptoms, hallucinations, and delusions) by factor analysis, we found an association between rs434473 and the negative symptom subgroup (Table 2). While our genetic data is statistically significant, the p values of our data are only weakly positive. We characterized the LD block between the three *ALOX12 *SNPs in the total subjects, using the pairwise D' values (Figure 1). The D' values from rs4730775 to rs32024233 and from rs37795488 to rs17132543 ranged over 0.99, indicating a strong LD between each pair of markers (Figure 1). This revealed an LD block, comprised of rs1126667, rs434473, and rs1042357.

**Table 2:** Genotype distribution of *ALOX12 *gene SNPs among schizophrenia patients in a Korean population.

SNP	Amino acid	Genotype	**Negative**^† ^**(+)** N = 168		**Negative**^† ^**(-)** N = 121		**χ**^**2**^	df	P
					
			Freq.	%	Freq.	%			
rs1126667	R261Q	G/G	41	24.4	36	29.8			
(Exon 6)		A/G	86	51.2	46	38.0	4.984	2	0.083
		A/A	41	24.4	39	32.2			

rs434473	S322N	A/A	41	24.4	37	30.6			
(Exon 8)		A/G	89	53.0	46	38.0	6.428	2	0.040*
		G/G	38	22.6	38	31.4			

rs1042357	T364T	C/C	41	24.4	36	29.8			
(Exon 8)		A/C	86	51.2	46	38.0	4.984	2	0.083
		A/A	41	24.4	39	32.2			

ALOX12, arachidonate 12-lipoxygenase; R, arginine; Q, glutamine; S, serine; N, asparagine; T, threonine; Freq, frequency; df, degree of freedom; p, probability value.

*The value is not significant after the Bonferroni correction (number of test = 18).

^†^Negative symptom subgroup identified by factor analysis of OPCRIT data.

Lipoxygenases are non-heme iron dioxygenase enzymes that insert molecular oxygen into free and esterified polyunsaturated fatty acids [11]. Research has suggested that *ALO*X12 might be involved with neurodegeneration [12]. Pratico et al. [13] showed that *ALO*X12 is higher in Alzheimer's disease, suggesting that it correlates with brain oxidative stress. In our data, rs1126667 and rs1042357 showed associations with schizophrenia in the recessive model. A/A carriers of rs1126667 and A/A carriers of rs1042357 had significantly higher odds ratios, of 1.60 (95% CI = 1.09-2.35), for schizophrenia. The SNP rs1126667 is a missense SNP that substitutes glutamine for arginine at amino acid position 261. This SNP is located in the lipoxygenase domain (amino acid position: from 124 to 655) in the ALOX12 protein. However, no research has yet shown a functional change in catalytic ability due to rs1126667. Although rs1042357 is synonymous, it can make an exonic splicing enhancer (5'- AGAAGC-3' and 5'- CAGAAG-3') with its A allele, whereas its C allele does not make one (genes.mit.edu/burgelab/rescue-ese). Thus, the A allele of rs1042357 in the lipoxygenase domain of the ALOX12 protein may lead to inaccurate splicing of pre-mRNA of the *ALOX12 *gene, which can produce an abnormal *ALO*X12 enzyme. In addition, rs434473 was associated with the negative symptom subgroup in OPCRIT items, which comprise slower activity, loss of energy/tiredness, loss of pleasure, and diminished libido (Table 2). There is evidence that 12-HPETE, the metabolite of arachidonate made by ALOX12 correlates with synthesis of catecholamine. High 12-HPETE inhibits protein kinase II [4]. In addition, research has shown protein kinase II is responsible for the phosphorylation of tyrosine hydroxylase, which is the rate-limiting step in the synthesis of (something is missing) [5]. The relatively less-phosphorylated tyrosine hydroxylase leads to reduced dopamine levels. In addition, 12-HPETE inhibits neurotransmitter release [6]. Thus, hypodopaminergia caused by decreased synthesis and release of dopamine might be present in deficit schizophrenia [14]. ALOX12 has been studied in connection with various malignancies, including colorectal cancer [11,12,15], breast cancer [16,17], prostate cancer [18-20], bladder cancer [21], and testicular cancer [22]. Research has shown that schizophrenia patients have an increased malignancy risk regarding colon cancer, marginally increased risk of breast cancer, and decreased risk of respiratory cancer [23]. ALOX12 might be one of the factors explaining the high risk of colorectal cancer in patients with schizophrenia. Thus, the relationship between ALOX12 and the higher incidence of colorectal cancer in schizophrenia patients needs future study. We conclude that rs1126667 and rs1042357 of the ALOX12 gene correlate with schizophrenia in a Korean population, and rs434473 correlates with negative symptoms in schizophrenia. These weak positives require additional replication study. These results suggest that future studies should regard ALOX12 as an important gene in the pathology of schizophrenia.

## Competing interests

The authors declare that they have no competing interests.

## Authors' contributions

Authors JWK and JHC designed and directed the entire project. HJK managed the literature searches and analyses. JKP carried out schizophrenia assessments and gave advice on patient selection. Author TK performed most of the statistical analyses and the genotyping and contributed substantially to the first draft of the manuscript. All authors contributed to, and have approved, the final manuscript
